# Human Platelet Lysate Can Replace Fetal Calf Serum as a Protein Source to Promote Expansion and Osteogenic Differentiation of Human Bone-Marrow-Derived Mesenchymal Stromal Cells

**DOI:** 10.3390/cells9040918

**Published:** 2020-04-09

**Authors:** Maria Karadjian, Anne-Sophie Senger, Christopher Essers, Sebastian Wilkesmann, Raban Heller, Joerg Fellenberg, Rolf Simon, Fabian Westhauser

**Affiliations:** Center of Orthopedics, Traumatology, and Spinal Cord Injury, Heidelberg University Hospital, Schlierbacher Landstraße 200a, 69118 Heidelberg, Germany; karadjian.maria@gmail.com (M.K.); annesophie.senger@gmail.com (A.-S.S.); c.essers@stud.uni-heidelberg.de (C.E.); sebastianwilkesmann@web.de (S.W.); raban.heller@outlook.com (R.H.); Joerg.Fellenberg@med.uni-heidelberg.de (J.F.); dr.rolf.simon@web.de (R.S.)

**Keywords:** osteogenic differentiation, population doublings, human mesenchymal stromal cells, fetal calf serum, human platelet lysate

## Abstract

Fetal calf serum (FCS) is frequently used as a growth factor and protein source in bone-marrow-derived mesenchymal stromal cell (BMSC) culture media, although it is a xenogenic product presenting multiple disadvantages including but not limited to ethical concerns. A promising alternative for FCS is human platelet lysate (hPL), which is produced out of human platelet concentrates and happens to be a stable and reliable protein source. In this study, we investigated the influence of hPL in an expansion medium (ESM) and an osteogenic differentiation medium (ODM) on the proliferation and osteogenic differentiation capacity of human BMSC. Therefore, we assessed population doublings during cell expansion, performed alizarin red staining to evaluate the calcium content in the extracellular matrix and determined the activity of alkaline phosphatase (ALP) as osteogenic differentiation correlates. The proliferation rate of BMSC cultured in ESM supplemented with hPL exceeded the proliferation rate of BMSC cultured in the presence of FCS. Furthermore, the calcium content and ALP activity was significantly higher in samples incubated in hPL-supplemented ODM, especially in the early phases of differentiation. Our results show that hPL can replace FCS as a protein supplier in cell culture media and does not negatively affect the osteogenic differentiation capacity of BMSC.

## 1. Introduction

Despite some limitations, fetal calf serum (FCS) is still the most common source of proteins and growth factors in cell culture media [1]. However, the composition of different batches of FCS varies crucially [2,3], making it necessary to evaluate different batches of FCS before ordering a new batch to cope with the batch-to-batch variability [1,4]; this evaluation takes time and resources [1]. The influence of FCS as a xenogenic compound of cell culture media on human cells is still not completely understood [1,5,6,7] as its composition differs substantially from the very complex human serum [8,9,10,11]. In addition, the conditions of FCS production regarding animal welfare have to be viewed critically, especially since production circumstances often remain unclear and information gathering is reported to be complicated [12]. This raises the question whether an alternative protein source standard in cell culture media is required.

The most common alternative to FCS is human platelet lysate (hPL) [1], which is produced from human platelets and contains proteins and growth factors necessary for, inter alia, in-vitro human bone-marrow-derived mesenchymal stromal cell (BMSC) expansion [13,14,15,16,17,18] and osteogenic differentiation [15,16,19]. hPL products can have smaller batch-to-batch variabilities by pooling multiple batches of platelet concentrates, hence extensive pre-testing becomes obsolete [17,20]. It has already been shown that hPL-supplemented expansion medium (ESM) augments the proliferation rates of BMSC [11,16,17,18,21,22,23,24,25,26,27,28,29], and does not affect BMSC properties as part of ESM [21]. Several studies evaluated BMSC differentiation potential after cell expansion in hPL-containing medium, while the differentiation medium itself contained only FCS as a supplement [17,18,22,23,28,29,30]. There have also been multiple studies reporting that hPL does not affect osteogenic differentiation [16,17,21,22,24,27,28,29,31] or even increases the osteogenic differentiation potential of BMSC [22,23,25,26]. However, it remains unclear how hPL as a part of an osteogenic differentiation medium (ODM) influences the kinetics of the osteogenic differentiation of BMSC. Furthermore, the necessary concentrations of hPL reported differ from 2% to 10% [18,20,25,26,29,30,31,32,33,34], pointing out that there is no established standard protocol for hPL supplementation in ODM for BMSC yet, although the concentration of serum supplement in media resulting in different concentrations of growth factors and other components of the serum influences cells in culture [1].

The aim of this study was to compare the performance regarding the proliferation rate and osteogenic differentiation of BMSC cultured in ESM and ODM containing different concentrations of hPL or FCS and to evaluate the impact of the protein supplement’s concentration on the osteogenic differentiation of BMSC. As most available studies on hPL assess its effects on mesenchymal stromal cells (MSC) during the expansion period, we focused on the effects of hPL as part of ODM on the kinetics of BMSC osteogenic differentiation by evaluating alkaline phosphatase (ALP) activity and calcium deposition as osteogenic differentiation markers at multiple time points.

## 2. Materials and Methods

The aims of this study were (i) to compare the impact of either FCS or hPL on the growth of BMSC under expansion conditions and (ii) to evaluate the osteogenic differentiation potential of BMSC cultivated in either FCS or hPL containing ODM. In order to compare the proliferation potential, population doublings of BMSC incubated in ESM with either 10% FCS or 10% hPL were calculated. For osteogenic differentiation experiments, the cells were incubated in ODM (containing either 1% or 10% of FCS or hPL, respectively) and measurement of ALP activity as well as alizarin red staining as markers for osteogenic differentiation were performed after incubation periods of 1, 7, 14 and 21 days (D1, D7, D14, D21). Twelve identical samples were analyzed for each assay at each time point.

BMSC were harvested from the bone marrow washouts of n = 10 patients undergoing surgery at the proximal femur at Heidelberg University Hospital. Prior to bone marrow harvesting, every patient gave written informed consent. The local ethics committee approved the study (S-443/2015) that was conducted according to the declaration of Helsinki in its present form. Cells derived from six male and four female patients with an average age of 50 years (range 29 to 73 years, median 46 years) were included in the study.

BMSC were isolated from bone marrow, cultivated according to previously published protocols and pooled in order to reduce inter-individual variability as published previously [35,36]. During cultivation and expansion, ESM containing either 10% FCS (Thermo Fisher Scientific, Dreieich, Germany; lot 42G2082K) according to our standard expansion protocol or 10% of commercially available hPL (PL BioScience, Aachen, Germany) according to the manufacturer’s instructions for high proliferation rates was used. The ESM was composed of 25 mM DMEM high-glucose (Thermo Fisher Scientific, Dreieich, Germany), Penicillin/Streptomycin 100 mg/L (Merck, Darmstadt, Germany), L-Glutamine 200 mM, MEM non-essential amino acids solution, 2-Mercaptoethanol 50 mM (all Thermo Fisher, Dreieich, Germany) and 4 µg/L fibroblast growth factor two (Abcam, Cambridge, United Kingdom).

BMSC were split after 72 hours of expansion and cultured for a further 96 hours before they were introduced to osteogenic differentiation conditions. Cells were then stained with trypan blue and counted in a Neubauer’s cell chamber. Population doublings within a total incubation period of seven days of expansion as a parameter for the proliferation rate were calculated using Formula (1).

(1) $$\mathrm{population}\;\mathrm{doublings}=\frac{\log N-\log N0}{\log2}$$

where N = cell number at the end of the expansion period and N0 = cell number at time point zero.

Population doublings of the passages were added in order to obtain cumulative population doublings.

For osteogenic differentiation, 35,000 BMSC were seeded in a well of a 24-well plate (Thermo Fisher Scientific, Dreieich, Germany) and incubated in ODM. The ODM was composed of 25 mM DMEM high-glucose with L-Glutamine (Thermo Fisher Scientific, Dreieich, Germany), Penicillin/Streptomycin 100 mg/L (Merck, Darmstadt, Germany), dexamethasone 0.1 µM (Sigma Aldrich, Steinheim, Germany), ascorbic acid-2-phosphate 2.5 mg/L (Sigma Aldrich, Steinheim, Germany) and β-glycerophosphate 10 mM (Merck, Darmstadt, Germany). The growth factor sources in ODM were either FCS or hPL, added in concentrations of either 1% (according to hPL’s manufacturer’s advice for differentiation culture) or 10% according to the well-known FCS standard concentration (Table 1). In the ODM containing hPL, 2 IU/mL heparin (PL BioScience, Aachen, Germany) were added according to the manufacturer’s instructions to avoid gel formation of the medium. The cells were incubated for up to 21 days at 37 °C, 5% CO_2_ following well-established protocols [36,37,38]. Media were changed twice per week.

ALP correlates with osteogenic differentiation of BMSC since it is produced during differentiation towards osteoblasts [37,38]. The cells were washed with 1x PBS (Thermo Fisher Scientific, Dreieich, Germany), then lysed in 1% Triton buffer (Sigma Aldrich, Steinheim, Germany). The lysates were freeze stored at −80 °C until the ALP activity measurement was conducted; after thawing at room temperature and short centrifugation, the lysates were added to a para-nitrophenylphosphat (p-NPP) (Sigma Aldrich, Steinheim, Germany) solution for 90 minutes. ALP hydrolyzes p-NPP to para-nitrophenol (p-NP), causing a change of color to yellow. The extinction of p-NP which corresponds to the ALP activity was measured photometrically at 405 nm with a reference wavelength of 490 nm using a MRX Microplate Reader (Dynatech Laboratories, Stuttgart, Germany). ALP activity was normalized to the total protein content as correlate for the cell number of the samples, determined using the Micro BCA Protein Assay Kit (Thermo Fisher Scientific, Dreieich, Germany) according to manufacturer’s instructions.

Alizarin red stains the calcium deposits in the extracellular matrix formed by the osteoblastic (precursor) cells [38]. The cells were washed with 1x PBS and then fixed in 70% ethanol (Carl Roth, Karlsruhe, Germany). After fixation, the ethanol was removed, the cells were washed with distilled water (Thermo Fisher Scientific, Dreieich, Germany), then stained with alizarin red (Waldeck, Münster, Germany) and washed again. Finally, the cells were incubated in 10% hexadecylpyridinium (Merck, Darmstadt, Germany) to dissolve the stained calcium. The extinction of the solution corresponds to the calcium content of the sample and was measured photometrically at 570 nm.

The experiments were performed on 12 experimental replicates per study group and two technical replicates per experimental sample. 

Statistical analyses were conducted with IBM SPSS Statistics (Version 25; IBM, Mannheim, Germany) and graphs were created using GraphPad Prism (Version 7, GraphPad Software, La Jolla, USA). Before further analysis, values were tested for normal distribution by a Shapiro-Wilk test. Normally distributed samples were tested with the unpaired T-Test; not normally distributed samples were tested using the Wilcoxon signed-rank test. Results were described as statistically significant for *p* < 0.05. Unless otherwise stated, differences mentioned in the text are non-significant. Values are shown as rounded means with standard deviation (SD) where applicable.

## 3. Results

### 3.1. Population Doublings

**Figure 1:**
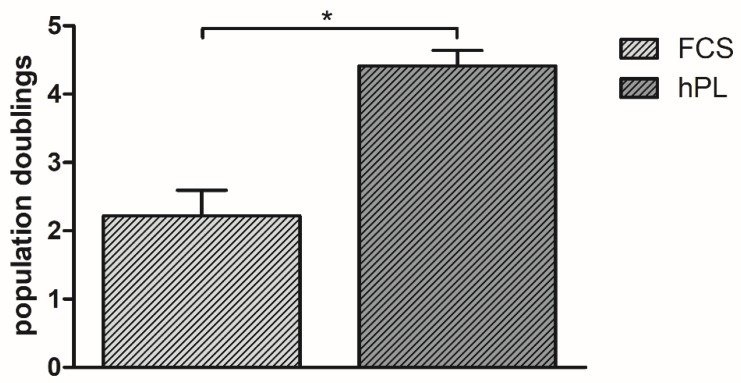
Population doublings of BMSC incubated in ESM. Values are presented as means with SD, * marks significant differences.

Cells cultured in 10% hPL-supplemented ESM had significantly (*p* < 0.01) more population doublings, with an average of 4.46 cumulative population doublings, than cells cultured in 10% FCS-supplemented ESM with an average of 2.22 cumulative population doublings (Figure 1).

### 3.2. Alkaline Phosphatase Activity

**Figure 2:**
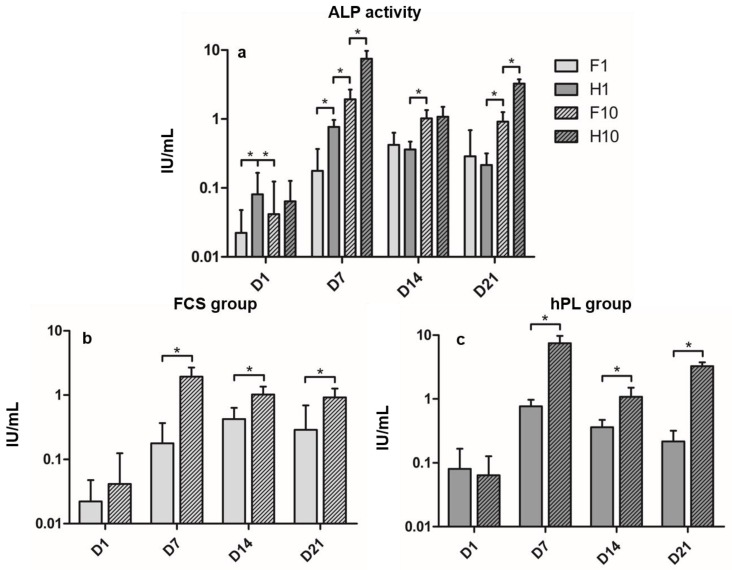
(**a**) Alkaline phosphatase activity during time of incubation in ODM in IU/mL of all groups. (**b**) ALP activity of FCS groups. (**c**) ALP activity of hPL groups. Values are shown as means with SD. * mark significant differences. D = day.

ALP activity kinetics differed among the four groups during the incubation period (Figure 2a). In the F1 group, ALP activity increased significantly from D1 to its maximum on D14, then remained on a stable level until D21, showing no significant differences between D14 and D21. The H1 group increased almost tenfold from D1 to D7, then decreased to D14 to further decrease until D21. The F10 group showed similar kinetics to the H1 group, but presented significantly different values to all time points. H10 showed its maximum ALP activity on D7, then significantly decreased to D14 to re-increase until D21. Differences between the F1 and H1 group were significant in the beginning of differentiation culture on D1 and D7, but showed no significant differences at D14 and D21. Differences between the H1 and the F10 group were significant at any time. Differences between the F10 and H10 group were significant on D7 and D21. In both hPL and FCS groups, cells incubated in the 10% supplemented media showed significantly higher ALP activity than the 1% groups on D7 to D21, but not on D1 (Figure 2b,c).

### 3.3. Alizarin Red Staining

**Figure 3:**
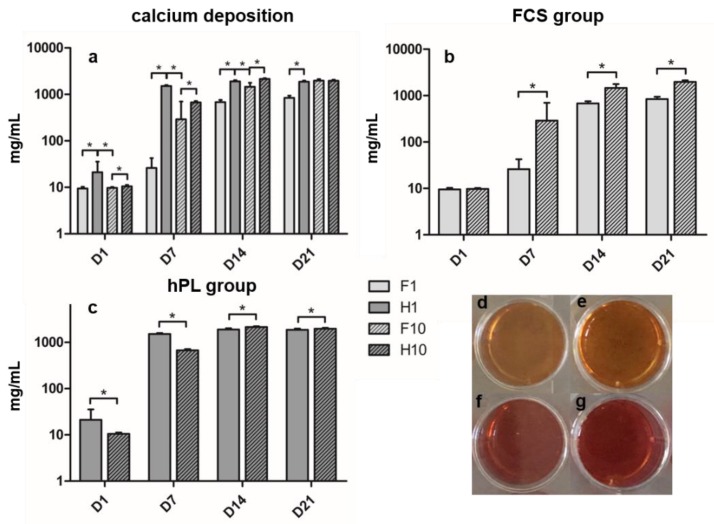
(**a**) Calcium content after alizarin red staining during time of incubation in ODM in mg/mL of all groups. (**b**) Calcium content of FCS groups. (**c**) Calcium content of hPL groups. (**d**–**g**) Cell layer in 24-well plate after alizarin red staining on D7. (**d**) F1. (**e**) F10. (**f**) H1. (**g**) H10. Values are shown as means with SD. * mark significant differences.

Calcium content increased in all groups over time of incubation. Calcium content in the hPL groups peaked on day 14, and the FCS groups showed maximum calcium content on day 21 (Figure 3a). During the whole differentiation period, the H1 group showed significantly higher calcium levels compared to the F1 group. H10 showed significantly higher calcium values at D1, D7 and D14 compared to F10, but lower values at D21 – however, the differences on D21 remained non-significant. When comparing H1 and F10, H1 showed significantly higher values from D1 to D14 and lower values on D21. When comparing the hPL groups (Figure 3c), H1 showed significantly higher calcium deposition than H10 on D1 and D7; this relation changed on D14 and D21 where H10 presented significantly higher values than H1. F1 presented the lowest values of all four groups on days seven to 21, significantly lower than the F10 group (Figure 3b).

## 4. Discussion

The aim of the study was to investigate whether hPL is a valid substitute for FCS in cell culture media for BMSC, especially in ODM, since FCS shows some limitations. For example, FCS composition differs essentially from human serum: Shanskii et al. [11] showed that hPL has significantly higher amounts of growth factors like IGF-1 (insulin-like growth factor 1) and PDGF (platelet-derived growth factor) than FCS. Despite the long-term use of FCS in cell culture, the effects of this xenogenic compound on human cells in culture remain unclear, also due to the fact that a majority of research on FCS dates from the past century [2,3,8]. Apart from the mentioned scientific concerns, also an ethical component has to be taken into account when discussing further usage of FCS: the European Food Safety Authority (EFSA) published a scientific opinion to, amongst others, answer the central question how to treat unborn livestock during slaughter, which is relevant for the procedure of FCS harvesting [39]. FCS is produced out of the blood collected by puncturing the heart of unborn, generally non-anesthetized cattle [20,40]. According to data obtained from Jochems et al. [12], cardiac puncture of the fetus begins between five and 30 minutes after the death of the dam and the bleeding procedure takes between two and five minutes. Another concern when it comes to ethical questions is the fact that FCS is mostly produced outside of the European Union; specifically, the exact origin of commercially available FCS often remains vague (e.g., origin “South America”) [12,39,40,41], making it almost impossible to trace specific FCS products.

hPL is generally produced out of a varying number of pooled human platelet concentrates [13,18,42,43,44,45] after platelet lysis by freezing—thawing cycles [20,43,44,45] or activation by thrombin [13,42] to liberate the substances required for cell culture that are stored in platelet granules [14,15]. However, there is no good manufacturing practice statement concerning the platelet lysis method in hPL production [13]. The hPL available for scientific purposes can be produced out of outdated platelet concentrates having no clinical use whilst being still suitable for (in-vitro research) cell culture [13,20,43]. Furthermore, the hPL composition creates a milieu closer to the physiological milieu of the human body than FCS does [8,9,10,11]. Therefore, hPL has favorable properties in terms of ethics and scientific practice and should seriously be considered as a new standard cell culture medium substitute.

To evaluate the impact of hPL on cell proliferation, we compared the expansion of BMSC in ESM supplemented with either 10% FCS or 10% hPL. We could show that BMSC expanded in hPL-supplemented ESM had more population doublings than their FCS-supplemented counterparts. However, the approach for evaluating cell proliferation in our study is a basic approach. For a more detailed evaluation, assays such as the 3H-thymidine incorporation assay or the 3-(4,5-dimethylthiazol-2-yl)-2,5-diphenyltetrazolium bromide (MTT) assay might provide a more detailed and standardized quantification of cell proliferation. The promoting effects of hPL on BMSC proliferation have been described before multiple times, in studies using the aforementioned more detailed assays; it has been shown that an enhanced proliferation of MSC is one of the main advantages in hPL supplementation of ESM [11,17,21,23,25,26]. Since our main focus was to assess the influence of hPL on the osteogenic differentiation of BMSC, we did not look into proliferation in greater detail. However, based on the assays performed, the results presented in this study confirm the results of previous studies that can be explained by the higher amount of growth factors in hPL, favoring cell proliferation and leading to a higher absolute growth factor amount in cell culture media when supplemented in the same concentration as FCS [11]. 

Many preliminary studies evaluated the effects of hPL-supplemented ESM on MSC in order to assess whether hPL changes MSC characteristics during expansion. Most of these studies evaluated the immunophenotype of the cells after expansion by analyzing cell surface markers with fluorescence-activated cell sorting (FACS): stem cell defining surface markers [46] were analyzed in almost any study; the consistent results were that expansion of MSC in hPL-supplemented ESM does not alter the expression of stem cell defining surface markers in comparison to MSC expanded in FCS-supplemented ESM [16,17,18,22,23,26,28,29,43,44,47]. Viau et al. [18] and Reis et al. [47] assessed the expression of further surface markers, revealing that the expression of some surface markers differs significantly between MSC expanded in hPL or FCS, but the majority of the markers are only mildly influenced by the source of protein supplement in ESM. Some studies additionally performed gene expression analyses of expanded MSC and revealed that, along with the results of protein expression of the investigated markers, the ESM’s protein supplement only mildly influences the gene expression pattern of MSC [29,43,44]. Fernandez-Rebollo et al. [44] performed DNA methylation analysis and revealed no significant differences between hPL- and FCS-expanded MSC on an epigenetic level. Viau et al. [18] evaluated BMSC morphology by immunofluorescence and FACS after expansion in hPL-containing ESM and revealed that these cells were smaller and less granular but more homogenous than their counterparts expanded in the FCS-supplemented ESM. Given the amount of studies that evaluated MSC characteristics after expansion in hPL-supplemented ESM and the extensiveness of this aspect, we did not perform any experiments regarding MSC characteristics after expansion in this study.

In order to evaluate hPL as a substitute for FCS in ODM, we compared the osteogenic differentiation of BMSC cultured in ODM supplemented with 1% hPL, 10% hPL, 1% FCS and 10% FCS, respectively. Alizarin red staining and ALP activity measurement were performed at four time points that cover all relevant steps of osteogenic differentiation in the used setting [48]. Osteogenic differentiation increased over time in all groups. The ALP activity of the BMSC in the hPL groups was higher than or as high as it was in the matching FCS groups. In addition, BMSC incubated in hPL-supplemented media presented a calcium content higher than or equal to the FCS groups at any time during cell culture. It has been shown previously that hPL can have favorable effects on the osteogenic differentiation of BMSC [22,23,26], providing every component required from serum necessary for cell culture [13,14,15,16,17,18,19] while respecting human serum composition [8,9,10]. However, our study is the first to evaluate the osteogenic parameters’ evolution under the influence of hPL over the differentiation period in two different concentrations, making it possible to track the influence of hPL on commonly used time points during osteogenic differentiation. This culture setting has been used regularly and covers the major steps of osteogenic differentiation by using the specified evaluation time points between one and 21 days of culture [36,37,38,49]: as described previously, the early phases in the differentiation of BMSC towards osteoblasts takes place from day five to 14 in culture and is characterized by ALP expression [48,50]. The calcification of the extracellular matrix produced during osteoblastic differentiation occurs from day 14 to 28 in culture [48,51,52]. 

The ALP activity of the hPL groups as well as the F10 group reach their maxima on day seven and decrease afterwards, so the kinetics of cells incubated in hPL- and FCS-supplemented ESM are similar. Only the F1 group increases until its maximum on day 14 and remains stable until day 21. Birmingham et al. [48] showed that osteoblastic cells show higher ALP activity than riper stages of the osteoblastic lineage. Considering the fact that H1, H10 and F10 groups reach their maxima earlier, it could be stated that osteogenic differentiation under those circumstances happens faster than under 1% FCS supplementation. There are no preliminary data concerning ALP kinetics of BMSC under hPL supplementation, as photometrical ALP activity measurement of hPL-supplemented BMSC was hardly performed before. Doucet et al. [21] described that ALP activity of BMSC in hPL-supplemented ODM was comparable to BMSC in FCS-supplemented ODM after 21 days, but no statement regarding ALP activity kinetics is available. Chevallier et al. [27] revealed that hPL can significantly up-regulate osteogenic genes like ALP in undifferentiated BMSC without exogenous osteogenic stimulation, whilst FCS-incubated BMSC reach the same level of gene expression only after one week of culture in ODM containing β-glycerophosphate and ascorbic acid. This finding could explain the superior ALP activity of the hPL groups, especially at the beginning of cell culture.

Focusing on the kinetics of extracellular calcium content, it is remarkable that both FCS groups increase to their maxima on day 21, whereas the hPL groups reach their maxima earlier, on day 14. Hoemann et al. [51] described that in-vitro mineralization normally occurs after two or three weeks of differentiation culture, hence both FCS and hPL groups show adequate kinetics, whilst hPL-incubated BMSC seem to differentiate faster than their FCS incubated counterparts. It is remarkable that no significant differences can be found between the H10 and the F10 group on day 21, implying that the final calcium content is similar in both groups while only the kinetics differ. Alizarin red staining is probably the most investigated parameter for osteogenic differentiation in hPL research, hence there is a lot of data available [16,22,23,25,26,27,29]. However, most studies performed a single time measurement and did not quantify the calcium content photometrically [16,26,27,29]. The studies that performed quantification of calcium content revealed that hPL-incubated BMSC show higher amounts of calcium than their FCS-incubated counterparts [22,23], which was confirmed in this study. 

Comparing FCS concentrations, it is remarkable that the commonly used concentration of 10% [1] seems to be necessary for adequate osteogenic differentiation as both ALP activity and calcium content remain higher (mostly to a significant extent) compared to 1% FCS-supplemented ODM. The hPL manufacturer recommended 1% hPL supplementation for BMSC differentiation, which is sufficient for a higher or comparable calcium content compared to 10% FCS (and 10% hPL). However, ALP activity in the H1 group remains beneath F10 and H10 groups at any time but D1. Interestingly, most studies determining hPL concentration in the context of osteogenic differentiation only determined calcium content [16,22,23,25,26,27,29,33,53]; only a few studies assessed ALP activity [18,21] and, therefore, did not detect the described discrepancy in ALP activity and calcium deposition. Only 10% hPL-supplemented ODM showed a constantly better or equal osteogenic differentiation compared to FCS. This discrepancy persists when comparing the absolute results of alizarin red staining and ALP activity measurement: in alizarin red staining, the hPL groups showed calcium content higher than or as high as the FCS groups while ALP activity varies more, especially when comparing H1 and F10 groups, where the F10 group presents higher ALP activity throughout differentiation from day seven onwards. For the pairs of H1 vs. F1 and H10 vs. F10, the previously described statement of superiority or equality of the hPL [16,17,21,22,23,24,26,27,28,31] is still valid.

According to our results and the data reported in the literature, hPL can be used as a supplement in ESM and ODM for BMSC, providing higher proliferation rates and higher or equal osteogenic differentiation potential. Our study was the first to evaluate the dynamics of ALP activity under the influence of two different concentrations of hPL in ODM, providing additional information about the kinetics of osteogenic differentiation under the influence of hPL. Furthermore, we reported a discrepancy of the impact of hPL in ODM on BMSC between two osteogenic differentiation parameters on a cellular/protein level, as there are hardly any studies that have evaluated two quantitative osteogenic differentiation parameters before. As we did not perform further assays evaluating osteogenic differentiation of BMSC on a genetic level, future studies should also evaluate osteogenic gene expression in order to obtain a more detailed impression of the impact of hPL in comparison to FCS on osteogenic differentiation in one and the same setting. 

The concentration of supplementation matters; in our case, only 10% hPL-supplemented ODM guaranteed an equal or higher osteogenic differentiation than the common approach using 10% FCS. However, we were the first study directly comparing two different concentrations of hPL in ODM, therefore further studies should follow in order to approach an optimum hPL concentration in ODM for BMSC. Considering the higher costs of hPL compared to FCS as well as the fact that the differences between the H10 and H1 groups were only detectable in the ALP activity assay, the use of hPL in smaller concentrations seems to be reasonable in order to avoid a significant increase of the budget necessary for cell culture experiments [54]. Based on the findings of this study, hPL should at least be considered as a potential alternative to FCS when analyzing osteogenic differentiation.

## 5. Conclusions

The main aim of this study was to evaluate the influence of hPL supplementation in different concentrations in an osteogenic differentiation medium on the osteogenic differentiation of BMSC, evaluated by alizarin red staining and ALP activity measurements. As already described in the literature before, we could show a positive impact of hPL supplementation in an expansion medium on BMSC population doublings, presenting a proliferation rate almost twice as fast as under FCS supplementation. Furthermore, we showed that osteogenic differentiation is not compromised, yet, favored by a hPL-supplementation in ODM in a concentration-dependent manner; the positive effect is most visible when hPL concentration is 10% and at the beginning of the differentiation period, implying an accelerated, but absolutely comparable osteogenic differentiation potential of human BMSC. 

## Figures and Tables

**Table 1:** Overview over the experimental groups.

Group	Protein Source and Concentration in ESM	Protein Source and Concentration in ODM
F1	FCS 10%	FCS 1%
F10	FCS 10%	FCS 10%
H1	hPL 10%	hPL 1%
H10	hPL 10%	hPL 10%
